# The long isoform of the *C. elegans* ELT-3 GATA factor can specify endoderm when overexpressed

**DOI:** 10.17912/micropub.biology.000748

**Published:** 2023-01-21

**Authors:** Gina Broitman-Maduro, Morris F. Maduro

**Affiliations:** 1 Department of Molecular, Cell and Systems Biology, University of California, Riverside, CA USA

## Abstract

The *C. elegans elt-3* gene encodes a GATA transcription factor that is expressed in the hypodermis and has roles in hypodermal specification and regulation of collagen and stress response genes. The gene encodes short and long isoforms, ELT-3A and ELT-3B respectively, that differ upstream of their DNA-binding domains. Previous work showed that ELT-3A can specify hypodermal cell fates when forcibly overexpressed throughout early embryos. We recently showed that the ELT-3B orthologue from the distantly related species *C. angaria* can specify endodermal fates when forcibly overexpressed in *C. elegans.* Here, we show that *C. elegans* ELT-3B can also specify endoderm.

**Figure 1. Gut specification network, END-3 and ELT-3 structures, and evidence that ELT-3B is sufficient to drive gut specification in C. elegans:**
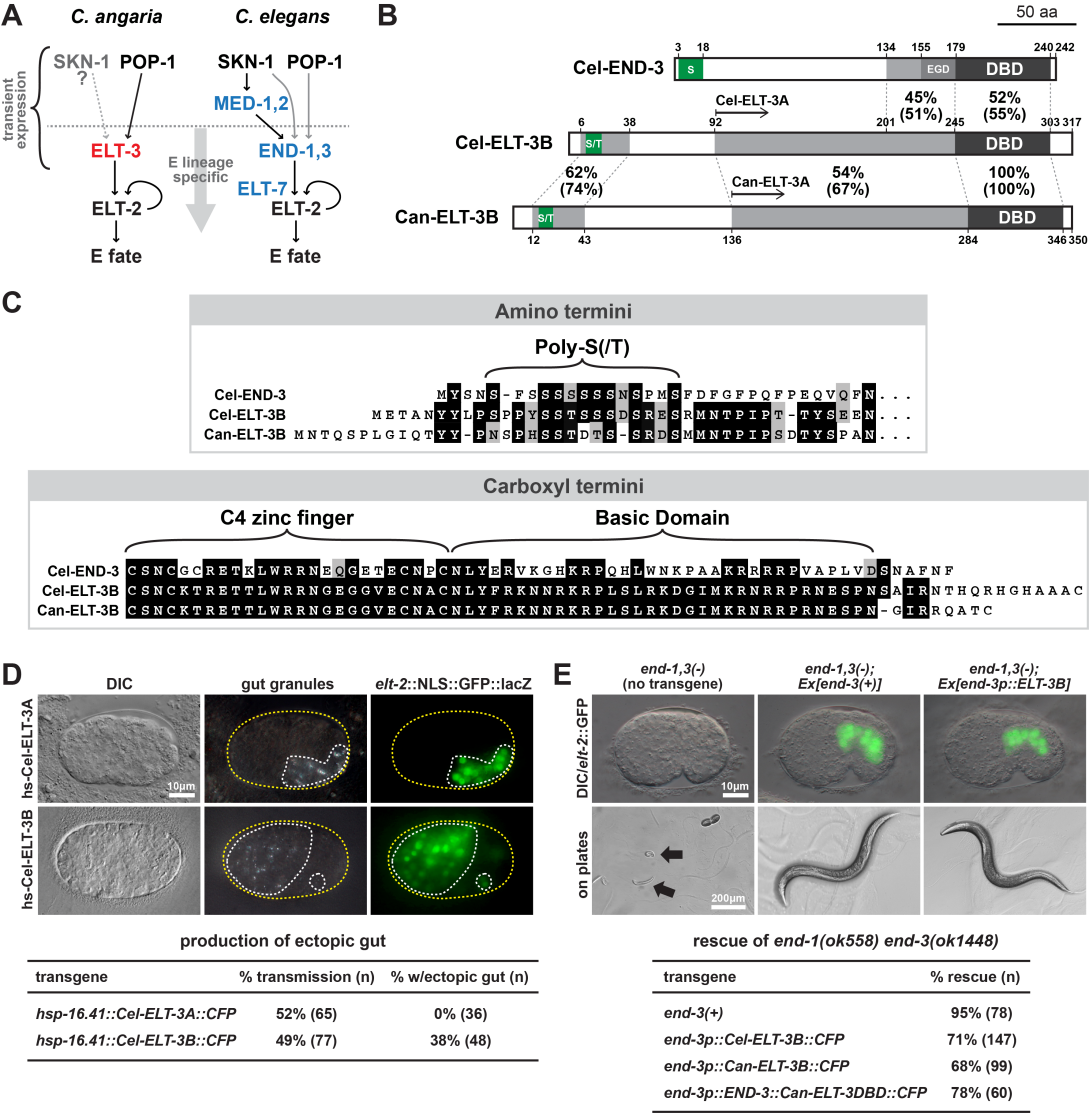
**A.** Simplified representations of the gene networks that specify gut in *C. angaria* and *C. elegans* , after a prior work (Broitman-Maduro et al., 2022). Factors below the horizontal dotted line act specifically in the E lineage. Factors upstream of ELT-2/-7 are transiently expressed. The common ancestor to both species is predicted to have used ELT-3 to specify the gut, while an expanded network of factors shown in blue acts in *C. elegans* . **B.** Comparison of ELT-3A/B from *C. angaria* and the ELT-3A/B and END-3 proteins from *C. elegans* . Percentages show identical amino acids (similar amino acids in brackets) across the regions identified by protein-protein BLAST (Altschul et al., 1990). The smaller ELT-3A isoforms start at amino acid positions 92 of Cel-ELT-3B and position 136 of Can-ELT-3B for *C. elegans* and *C. angaria* , respectively. Abbreviations: DBD, DNA-binding domain; EGD, Endodermal GATA Domain found in END-1 and END-3 orthologues (Maduro, 2020). **C.** Protein alignments of amino and carboxyl ends of Cel-END-3, Cel-ELT-3, and Can-ELT-3. Extended homology exists between the amino ends of the two ELT-3B orthologues, however Cel-END-3 shares only a similar Poly-S region with these. In the carboxyl ends which contain the DNA-binding domains (DBDs), those of Can-ELT-3 and Cel-ELT-3 are essentially identical, while Cel-END-3 shares only 52% identity (55% similarity) with Cel-ELT-3. Alignments were performed manually, assisted by BLASTP results (Altschul et al., 1990). **D.** Forced overexpression of Cel-ELT-3B, but not Cel-ELT-3A, promotes ectopic specification of gut. Left column: Representative DIC images of arrested transgenic embryos following heat shock (hs). Middle column: Polarized light showing presence of gut granules (white dots) in the same embryos as in the first column. In the case of hs-ELT-3B, granules are widely dispersed. Right column: Expression of integrated *elt-2* reporter transgene in the same embryos as in the first column. In the middle and right-hand columns, the eggshells have been outlined in a dotted yellow line, and groups of gut cells, shown by both gut granules and *elt-2* expression, have been outlined with dotted white lines. All images are at the same scale. Bottom: Table of results of heat shocked embryos. Gut was scored as ectopic if there were more than 40 *elt-2-* expressing nuclei present. Embryos of hs-Cel-ELT-3A generally had 20 or fewer *elt-2* -expressing nuclei. **E.** Rescue of the embryonic/larval lethality and gut specification defects of *end-1(ok558) end-3(ok1448)* double mutants. Images show *end-1,3(-)* without or with rescue by extrachromosomal arrays carrying either *end-3(+)* (second column) or an *end-3promoter::ELT-3B::CFP* transgene (third column). Top row: DIC with GFP overlay showing expression of integrated *elt-2promoter::NLS::GFP::lacZ* reporter *wIs84* in rescued comma-stage embryos. Bottom row: Representative arrested L1 larvae (arrows) or rescued transgenic adults on agar. Images across each row are at the same scale as the leftmost image. Bottom: Table showing rescue of *end-1,3(-)* lethality by various transgenes. Rescue was scored as positive if transgenic animals survived past the L3 stage.

## Description

The intestine (gut) is clonally derived from the embryonic E blastomere in *C. elegans* (Sulston et al., 1983). A cascade of GATA transcription factors, MED-1,2 → END-1,3 → ELT-2,7, drives specification and commitment to the endodermal fate downstream of maternally provided SKN-1/Nrf and POP-1/TCF (Fig. 1A) (Bowerman et al., 1992; Lin et al., 1995; Maduro et al., 2005; Maduro, 2017; Maduro et al., 2001; Maduro and Rothman, 2002; McGhee, 2007; McGhee et al., 2009; Sommermann et al., 2010; Zhu et al., 1997). The MED-1,2 factors function in specification of both E and its sister cell MS; hence, the E lineage-specific portion of the network starts with expression of *end-3* after E is born and then *end-1* shortly after (Baugh et al., 2003; Maduro et al., 2015; Maduro et al., 2001). The transient expression of the MED and END factors leads to production of the main intestinal organ identity factor ELT-2, which along with enforcement by ELT-7, drives continued differentiation of the intestine and maintenance of intestinal integrity (Dineen et al., 2018; Fukushige et al., 1998; Sommermann et al., 2010; Wiesenfahrt et al., 2015).

Among sequenced *Caenorhabditis* species, only those in the Elegans supergroup encode MED, END and ELT-7 factors (Maduro, 2020). Among all nematodes of Clade V that includes *Caenorhabditis* , a core set of four classical GATA factors is found, namely ELT-1, ELT-2, ELT-3 and ELT-5 (EGL-18 in *C. elegans* ) (Eurmsirilerd and Maduro, 2020).

We recently proposed that a much simpler zygotic gut specification network consisting of ELT-3 → ELT-2 is found in *C. angaria* (Fig. 1A) (Broitman-Maduro et al., 2022). In this species, which is outside of the Elegans supergroup, RNAi or mutation of *Can-elt-3* results in a penetrant absence of gut, similar to mutation of *end-1* and *end-3* together in *C. elegans* (Broitman-Maduro et al., 2022; Owraghi et al., 2010). POP-1/TCF acts upstream of *Can-elt-3* , and *Can-elt-2* downstream, but a role for a Can-SKN-1 orthologue could not be found (Broitman-Maduro et al., 2022). The role of *elt-3* as a zygotic endoderm specification factor is likely to be the ancestral state in the genus, as a proposed stem species, *C. monodelphis* , is an outgroup to both *C. elegans* and *C. angaria* and also has an *elt-3* orthologue that is expressed in the early E lineage upstream of its *elt-2* orthologue (Broitman-Maduro et al., 2022; Slos et al., 2017). It is formally possible that there are more genes in the network in *C. angaria* and other species, however expression of only *Can-elt-3* and *Can-elt-2* is sufficient to recapitulate the entire E lineage portion of the gut network when introduced into a *C. elegans elt-2; elt-7 end-1 end-3* quadruple mutant (Broitman-Maduro et al., 2022).

While *Can-elt-3* is required for endoderm specification in *C. angaria* , no endodermal specification role is apparent for *C. elegans elt-3* . A null mutation in *Cel-elt-3* results in no apparent developmental phenotype (Gilleard and McGhee, 2001). Second, loss of *Cel-elt-3* does not synergize with loss of *end-1* , *end-3* or *elt-7* , to cause increased defects in gut specification (Broitman-Maduro et al., 2022). Studies of *elt-3* have implicated roles in hypodermal specification (Gilleard and McGhee, 2001; Shao et al., 2013) and in regulation of cuticle collagen genes and other genes involved in response to oxidative stress (Gilleard and McGhee, 2001; Hu et al., 2017; Mesbahi et al., 2020; Shao et al., 2013). Curiously, single-cell RNA-seq studies recovered low levels of *elt-3* transcripts in the early E lineage, though not in the E cell itself (Hashimshony et al., 2015; Tintori et al., 2016). However, we failed to observe any such expression in intact embryos by single-molecule inexpensive fluorescence *in situ* hybridization, or smiFISH (Broitman-Maduro et al., 2022; Tsanov et al., 2016). Hence, there has been no reason to hypothesize a role for *C. elegans elt-3* in endoderm specification.

The *elt-3* genes in *C. angaria* and *C. elegans* encode at least two ELT-3 isoforms, one short (ELT-3A), and one long (ELT-3B) (Broitman-Maduro et al., 2022; Li et al., 2020). In *C. angaria* , smiFISH probes that detect mRNA for both isoforms reveal *elt-3* transcripts in the early E lineage and later in the hypodermal lineages; the hypodermal expression resembles previously known expression of *C. elegans elt-3* (Broitman-Maduro et al., 2022; Gilleard et al., 1999). In contrast, mRNAs for the long isoform of *Can-elt-3* were detected only in the early E lineage, suggesting that Can-ELT-3B is endoderm-specific. Consistent with this, forced expression of Can-ELT-3B in early *C. elegans* embryos, but not Can-ELT-3A, is sufficient to drive endoderm specification (Broitman-Maduro et al., 2022). Early work on *elt-3* found that overexpression of Cel-ELT-3A throughout early embryos is sufficient to promote specification of hypodermal fates (Fukushige et al., 1998; Gilleard and McGhee, 2001). In those studies, only the shorter ELT-3A isoform was known at the time, and hence overexpression of ELT-3B was never tested.

END-3 is at the top of the E-specific part of the gut specification network in *C. elegans* and is the most comparable to ELT-3B in *C. angaria* . Cel-END-3, Cel-ELT-3B, and Can-ELT-3B have similar overall structures (Figs. 1B,C). All three share a GATA type DNA-binding domain (DBD) at their carboxyl ends, consisting of a C4 GATA type zinc finger and basic domain (Broitman-Maduro et al., 2022; Maduro, 2020). Whereas the DBDs of Can-ELT-3B and Cel-ELT-3B are identical, the DBD of Cel-END-3 is more diverged, sharing only 52% identity and 55% similarity with Cel-ELT-3B. The long isoforms of ELT-3 share a short region of homology at their amino termini that includes a Poly-S/T low-complexity region. A similar Poly-S region was previously observed in the amino termini of END-like endodermal GATA factors, including END-3 (Maduro, 2020). The shorter Cel-ELT-3A and Can-ELT-3A isoforms likely result from transcriptional initiation farther downstream and therefore lack the amino terminal regions found in the long isoforms.

The endodermal GATA factors MED-1,2, END-1,3, and ELT-2,7, have the ability to individually promote widespread gut specification when forcibly expressed throughout early embryos (Fukushige et al., 1998; Maduro et al., 2005; Maduro et al., 2001; Owraghi et al., 2010; Sommermann et al., 2010; Zhu et al., 1998). To test whether *C. elegans* ELT-3B can specify endoderm when forcibly overexpressed, we constructed heat shock (hs) promoter fusion transgenes *hs::Cel-ELT-3A::CFP* and *hs::Cel-ELT-3B::CFP* and introduced these separately as extrachromosomal arrays into a strain carrying an integrated *elt-2promoter* ::NLS::GFP::lacZ reporter, *rrIs1* , which marks differentiated intestinal cells (Fukushige et al., 1998; Kostic and Roy, 2002; Mello et al., 1991). We obtained several transgenic lines and chose the best transmitting line for further study in each case. We heat shocked gravid hermaphrodites on plates at 34°C for 20 min. After 2 h at 21°C, we observed widespread nuclear CFP, confirming overexpression, which allowed us to also measure the transmission frequency of the arrays (~50% for both). We allowed embryos to develop for an additional 10 h and examined them for the presence of gut granules (a marker of differentiated endoderm) and expression of *elt-2* . As shown in Fig. 1D, we observed ectopic gut cells in 38% (n=48) of embryos from the *hs::Cel-ELT-3B::CFP* strain, similar to prior results with similarly overexpressed Can-ELT-3B::CFP (Broitman-Maduro et al., 2022). Heat shock of the *hs::Cel-ELT-3A::CFP* transgene strain failed to produce embryos with ectopic endoderm, consistent with prior results (Fukushige et al., 1998; Gilleard and McGhee, 2001).

We next tested for the ability of forced early E lineage expression of ELT-3B-derived transgenes to restore gut specification to a genetic background that is unable to do so (Fig. 1E). We used strain MS1094 in which the *end-1(ok558) end-3(ok1448)* genetic background is rescued by an extrachromosomal array carrying *end-3(+)* and which is marked by an *unc-119promoter::mCherry* reporter (Owraghi et al., 2010). In MS1094, 95% (n=78) of transgenic embryos were rescued to viability, scored as survival to young adult stage, while embryos lacking the array arrested as embryos or larvae lacking gut. We replaced the *end-3(+)* array with one carrying an *end-3promoter* ::Cel-ELT-3B::CFP transgene, and found rescue of 71% of transgenic embryos (n=147) to viability. To compare with *C. angaria* ELT-3B, we introduced an *end-3promoter* ::Can-ELT-3B::CFP transgene and obtained a similar rescue of 68% (n=99). Representative rescued animals are shown in Fig. 1E; the rescued embryos are strains into which an *elt-2promoter::NLS::GFP::lacZ* reporter ( *wIs84* ) has been crossed in. To test whether the reduced efficiency of rescue was a function of the DBD or upstream regions, we tested whether replacing the DBD of END-3 with that of Can-ELT-3, but leaving the upstream amino portion of END-3 intact, would improve rescuing ability. Such an *end-3promoter* ::END-3::Can-ELT-3DBD::CFP transgene array was able to rescue 78% (n=60) of transgenic embryos to viability, however rescue among the ELT-3-derived transgenes was not significantly different ( *p* > 0.2, pairwise χ ^2^ -tests). Hence, the reduced efficiency of rescue could be attributed to differences in the DNA-binding domains between ELT-3 and END-3. The results nonetheless show that the ELT-3B isoforms from *C. elegans* and *C. angaria* can rescue gut specification most of the time in an *end-1,3(-)* genetic background when expressed in the early E lineage.

The ability of *C. elegans* ELT-3B to activate gut specification is an unexpected result, given that *Cel-elt-3* has no known role in the endoderm. Endoderm-specifying ability is not a general property of GATA factors, as prior work showed that overexpression of ELT-3A or ELT-1 does not promote gut specification (Fukushige et al., 1998). Similarly, forced expression of ELT-3A or ELT-1 in the early E lineage cannot rescue gut specification mutants, although such transgenes with ELT-2 or ELT-7 can (Dineen et al., 2018; Wiesenfahrt et al., 2015). We note that in these experiments, rescue was tested in both *end-1 end-3* double mutants and *elt-7 end-1 end-3* triple mutants and using either the *end-1* and *end-3* promoters, with similar results; here we have tested for rescue of only the *end-1 end-3* double mutant background, using transgenes driven by the *end-3* promoter. Qualitatively these assays are very similar, since all gut specification is already abolished in an *end-1 end-3* double mutant, and either *end* promoter drives similar early E lineage expression, although *end-3* is activated slightly earlier (Baugh et al., 2003; Maduro et al., 2005; Owraghi et al., 2010; Zhu et al., 1997).

Why does *C. elegans* ELT-3B have gut-promoting activity? The results raise the possibility that ELT-3B plays a cryptic role in gut specification, or that it activates some aspects of intestinal function, perhaps under certain conditions. The latter might explain the reported genetic requirement for *elt-3* in longevity and stress response (Budovskaya et al., 2008; Hu et al., 2017; Mesbahi et al., 2020). Whether such functions for ELT-3 directly involve expression in the adult intestine has been controversial (Budovskaya et al., 2008; Tonsaker et al., 2012). More may be known after the spatiotemporal expression pattern of the long isoform (specifically) is determined.

Another question raised by these results is how the extended amino region of ELT-3B converts ELT-3A into a ‘gut specifier’ as opposed to a ‘hypodermal specifier’, since both isoforms have the same DNA-binding domain. The amino termini of ELT-3B and END-3 contain domains enriched in serine, a feature of GATA factors that is found among those that specify endoderm (Maduro et al., 2005; Maduro, 2020). Serine tends to be enriched in protein regions that are intrinsically disorganized (Uversky, 2015). In transcription factors, such regions could help promote gene expression through phase separation to establish rapid initiation (Hnisz et al., 2017; Sabari et al., 2018). The upstream regions of END-3 and ELT-3B might therefore enable rapid initiation of gut specification through a state change, establishing protein-protein interactions at the *elt-2* promoter that are not possible with the shorter ELT-3A isoform. With distinguishable functions encoded in isoforms of the same *C. elegans* GATA factor, these structure-function questions can now be studied.

## Methods

**Strain maintenance and transgenesis**

*C. elegans* animals were grown on NGM plates using standard conditions at 20°C-23°C. Strains are listed in the Strain Table. Transgenics were made by microinjection which generates multicopy extrachromosomal arrays (Mello et al., 1991).

**Plasmids and cloning**

We ordered the synthesis of the ELT-3B coding region from Integrated DNA Technologies, Inc. Using Gibson assembly we constructed heat shock and *end-3-* derived recombinant plasmids analogous to methods described previously (Broitman-Maduro et al., 2022; Gibson et al., 2009). Cloning of plasmids is described in the Plasmid table. Primers used in cloning are described in the Primer table. Coding sequences for Cel-ELT-3 were obtained from WormBase WS285 as K02B9.b (ELT-3B) and K02B9.a (ELT-3A), respectively. We confirmed the predicted coding regions for Can-ELT-3A/B by sequencing RT-PCR products obtained from mixed-stage *C. angaria* embryos using first-strand primer GBM144 and SL1 primer GBM176 with reverse primers GBM118 for ELT-3A and GBM177 for ELT-3B. A minor splice difference was observed in the first intron that resulted in a change of three amino acids from the previously predicted coding region, which we repaired as described below. The *C. angaria elt-3* gene and coding DNA sequences have been deposited in GenBank (Accession number OP850274). Reporter transgenes for *unc-119* were from prior work (Coroian et al., 2005; Owraghi et al., 2010). Plasmids and cloning information for these are available on request.

**Imaging and image processing**

Animals were imaged on an Olympus BX51 upright microscope equipped with DIC and fluorescence using a Canon 77D digital camera and LMscope adapter ( www.lmscope.com ). Images were adjusted for color and contrast with Adobe Photoshop CS5.1. The figure was assembled with Adobe Illustrator CS5.1.

## Reagents

**Strain table**

**Table:** 

**Strain**	**Genotype**
MS1094	*end-1(ok558) end-3(ok1448) V; irEx498 [end-3(+), unc-119promoter::mCherry]*
MS1127	*end-1(ok558) end-3(ok1448) V; wIs84 [elt-2promoter::NLS::GFP::lacZ* , *rol-6 ^D^ ] X* ; *irEx498 [end-3(+), unc-119promoter::mCherry]*
MS2599	*unc-119(ed4) III; end-1(ok558) end-3(ok1448) V; irEx804 [end-3promoter::END-3::Can-ELT-3_DBD::CFP* , *unc-119promoter::NLS::YFP::lacZ* , *unc-119(+)]*
MS2639	*end-1(ok558) end-3(ok1448) V; irEx819 [end-3promoter::Cel-ELT-3B::CFP* , *unc-119promoter::NLS::CFP]*
MS2640	* rrIs1 [elt-2::NLS::GFP::lacZ, unc-119(+)] X; irEx820 [hsp-16.41promoter::Cel-ELT-3B::CFP, rol-6 ^D^ ] *
MS2641	* rrIs1 [elt-2::NLS::GFP::lacZ, unc-119(+)]] X; irEx821 [hsp-16.41promoter::Cel-ELT-3A::CFP, rol-6 ^D^ ] *
MS2643	*end-1(ok558) end-3(ok1448) V; irEx822 [end-3promoter::Can-ELT-3B::CFP* , *unc-119promoter::NLS::CFP]*
MS2644	*end-1(ok558) end-3(ok1448) V; wIs84 [elt-2promoter::NLS::GFP::lacZ* , *rol-6 ^D^ ] X* ; *irEx819 [end-3promoter::Cel-ELT-3B::CFP* , *unc-119promoter::NLS::CFP]*

**Plasmid table**

**Table:** 

**Plasmid**	**Genotype**	**Description**
pGB644	*hsp-16.41promoter::Cel-ELT-3B::CFP::end-3_3’UTR*	constructed by Gibson Assembly of PCR products from the following reactions: GBM90/GBM178 on pPD49.83 (hsp-16.41 vector), GBM179/GBM158 from IDT-synthesized intronless coding region for ELT-3B, GBM159/85 to amplify FP::end-3_3’UTR from pGB596 (Broitman-Maduro *et al.* 2022) into pBluescript KS- digested with *Hind* III and *Bam* HI
pGB645	*hsp-16.41promoter::Cel-ELT-3A::CFP::end-3_3’UTR*	constructed by Gibson Assembly of PCR products from the following reactions: GBM90/GBM180 on pPD49.83, GBM181/158 from IDT-synthesized intronless coding region for ELT-3B, GBM159/GBM85 on pGB596 into pBluescript KS- digested with *Hind* III and *Bam* HI
pGB648	*end-3promoter::Cel-ELT-3B::CFP::end-3_3’UTR*	constructed by Gibson Assembly of PCR products from the following reactions: GBM80/GBM196 to amplify the *end-3* promoter from pGB595 (Broitman-Maduro *et al.* 2022), GBM197/GBM158 from IDT-synthesized intronless coding region for ELT-3B, GBM 159/85 from pGB596 into pBluescript KS- digested with *Hind* III and *Bam* HI
pGB649	*end-3promoter::Can-ELT-3B::CFP::end-3_3’UTR*	constructed with Q5 BaseChanger Kit (New England Biolabs) with CANelt-fixF and CANelt-fixR on pGB618 (Broitman-Maduro *et al.* 2022)

**Primer table**

**Table:** 

**Primer**	**Sequence (5’ to 3’)**
GBM80	CGGCCGCTCTAGAACTAGTGCATCCAATTTAGTGTATATTTATTTCC
GBM83	CTTTACTCATGCATGTGGCTTGTCTTCTG
GBM85	CGAGGTCGACGGTATCGATAAGCTCTTTCCACTATAGTC
GBM90	CGGCCGCTCTAGAACTAGTGGATCACCAAAAACGGAAC
GBM118	CTTTACTCATAGCCATTCGCTTTTTGACAAGTGG
GBM144	CAAATTGAATTTTGATGACATTGCACGGC
GBM158	CTTTACTCATACAAGCAGCTGCGTGTCCATGTCTCTG
GBM159	AGCTGCTTGTATGAGTAAAGGAGAAGAACTTTTCAC
GBM176	GGTTTAATTACCCAAGTTTGAG
GBM177	TTGATACTGTTGCTCAAATTGATGATCTTG
GBM178	CAGTTTCCATGGATCCCGATGAGGATTTTC
GBM179	ATCGGGATCCATGGAAACTGCCAACTATTACTTAC
GBM180	AGTCCTTCATGGATCCCGATGAGGATTTTC
GBM181	ATCGGGATCCATGAAGGACTCTCAACTTTCCG
GBM196	CAGTTTCCATGTTTATACTTTGAATGAGAATGC
GBM197	AAGTATAAACATGGAAACTGCCAACTATTACTTACC
CANelt-fixF	ATGATGAATACACCAATTCCAAGTG
CANelt-fixR	GCTATCACGTGATGATGTATCAG
